# American Academy of Dental Science

**Published:** 1902-03

**Authors:** 

**Affiliations:** American Academy of Dental Science


### AMERICAN ACADEMY OF DENTAL SCIENCE.

The thirty-fourth annual meeting and banquet of the American Academy of Dental Science was held at Young's Hotel, Boston, on Wednesday evening, November 13, 1901, at six o'clock. Previous to the dinner, the regular business of the organization was transacted, and the following-named officers were chosen for the ensuing year:

President, Frederick Bradley, D.M.D., Newport, R. I.; Vice-President, Forest G. Eddy, D.M.D., Providence, R. I.; Recording Secretary, Walter E. Decker, D.D.S., Boston; Corresponding Secretary, Frederick E. Banfield, D.M.D., Boston; Treasurer, William Y. Allen, D.D.S., Boston; Librarian, Herman G. Hichborn, M.D., D.D.S., Boston; Editor, Charles H. Taft, D.M.D., Boston.

*Executive Committee.*—Thomas Fillebrown, M.D., D.M.D.; Stephen G. Stevens, D.D.S.; William H. Potter, D.M.D.

S. M. Macvane, Ph.D., McLean Professor of Ancient and Modern History in Harvard University, was the guest and principal speaker of the evening.

*President Pond.*—It is my very pleasant duty to welcome you here to this thirty-fourth annual meeting. I remember very well at our twenty-first annual meeting Dr. Elisha Tucker was present, and he spoke of some of the early work of the Academy, and its struggles, and congratulated us upon having reached the age of twenty-one years. He expressed a hope that, having become a full-grown man, twenty-one years of age, we would continue and do a man's work.

I think this last year we have continued in a way that would please him. We have had our regular meetings, with one or more papers at every meeting, and discussions on them. The membership continues at its height, and the treasurer has been able to meet all bills. We have certainly had a prosperous year and done good work.

Dr. Tucker spoke of its meetings, and efforts to do away with little petty jealousies that seemed to be alive at that time, of trying to help each other, and trying to give professional standing to the dentists. It seems to me that we, individually and collectively, have yet a good deal of work to do, something that we can do, something that we ought to do. One of the first things is dental education. We are constantly hearing of the requirements for admission to the schools. At a recent meeting of the Twentieth Century Club, President Eliot said that the schools of divinity, medicine, and law have now reached a point where they require a college education for admission. They apparently have reached the highest point possible. The discussion continued in regard to the education of school-teachers. Normal schools are established like our professional schools, and formerly many of the applicants have not even had a high school education. Now they are calling for a college education as a requisite for admission.

What are we going to do about this? How long can we continue with our present standards of education? We cannot stand still. If the others are advancing—and we know they are—they will leave us behind, unless we advance with them. We must keep up with this standard set by the other professions. Perhaps we can do something the coming year, individually or collectively. The dental schools were brought into existence by the profession. Now, without doubt, if the dental profession call for a higher standard of education, work for it, and support it, the dental schools will meet that demand. If we are to stand with the other professions, we must equal them.

This leads almost directly to another subject, and that is our laws. The only justification of a law regulating the practice of dentistry, or any other practice, is to take it on the ground that it is a protection to the people. Laws are not passed intentionally or directly to benefit you or me, but to protect the people. Neither are they intended to work a hardship upon any one. The practical application of our laws to-day certainly works a hardship on the dentists. If any member of this Academy, certainly a practitioner in good standing, should have to leave the State, what is he going to do? He cannot practise. The average man, after a few years' practice, cannot pass the examinations. Of course, we can become day laborers, but we cannot continue to be dentists. In Dr. Andrews's paper he mentions a man who could not pass the examination papers, although he had been a successful practitioner for many years, and was a regular graduate of a dental college; and a member of the examining board in a neighboring State was unable to pass the examinations of our board recently. I do not suppose that we of this society would fare much better if we should try. That is a thing that we should work against: it is entirely wrong, and we should try to remedy it.

That comes back again to the question of education. When anything is said about accepting the examinations of one State in another, we have to meet the difficulty of the difference in the standing of schools. One State feels that the examinations of the others are not their equal, and they are not accepted. It seems to me that we can do, and ought to do, considerable work in the direction of equalizing the standard of the schools and the examining boards, so that we may not cease to be dentists if we happen to cross a State line.

There is another point that was spoken of at that time. I was looking over the records of some of the earlier meetings, and they seem to have interested themselves very much in what might be called the proprieties of professional life, the relation of the dentist to the general public; not entirely to the patients, but to the public at large,—our standing with the general public. I remember when I was a student, twenty years ago, it was the aim and ambition of every young man, in starting for himself, not only to get a practice, but to observe the outward proprieties of practice. This idea was impressed upon us by our teachers. It was the desire and aim to be established in a house with professional surroundings: that seemed to be the expected thing then. It seems to me there is to-day inclination on the part of our young men, young men who are graduates of good schools, to go perhaps into the business section of the city and take a room not specially adapted to dental practice, a room surrounded by that which is certainly not professional; perhaps on one side Dr. Humbug No. 1, who tells your fortune from the stars; and on the other side Dr.

Humbug No. 2, who has an electric belt, and cures everything. This is not at all professional. The rooms are perhaps fitted up with two or three slight partitions, the same as you see manicure parlors, chiropodists, magnetic healing apartments, etc. And the young men start in practice in that way. It seems to me that that is something that we can perhaps work against and possibly do something to change the ideas and tendencies of many of our young men. If a man is seen on the street in jumper and overalls and with a dirty old hat on, and he is offered a job as a laborer, I do not believe he would feel that it was anything more than he should expect. A gentleman told me that in England a man could not succeed in practice unless he wore glasses and evening dress all day. Perhaps it is carried a little too far there, but it seems to me that our young men should be interested and given a tendency to different things, so they will observe the proprieties of a profession. I fail to see, if a man is in these surroundings, how he can expect, and how we can expect, the outside public to distinguish that one as reputable and the other one as a humbug; and it seems to me that if these things are to continue we can hardly expect to be called professional. We may be called professional men in the same sense that a boot-black is called "professional."

This, as I say, is our thirty-fourth annual meeting. Following the usual custom of our annual meetings, in which we depart a little from our regular dental papers, and make it an enjoyable occasion, having a paper or talk by somebody on some special subject not strictly dental, but of great interest to us, we have with us to-night Professor S. M. Macvane, Ph.D., McLean Professor of Ancient and Modern History in Harvard University. It gives me great pleasure, gentlemen, to present Professor Macvane, of Harvard.

(For Professor Macvane's paper, see page 156.)

DISCUSSION.

*Dr. Fillebrown.*—I would like to ask a question or two on this point. The subject interests me very much.

First. How much have the entrance examinations of our colleges been increased within the last twenty-five years? That is, how much more must a man know now to enter Harvard than was required twenty-five years ago?

Second. Whether the scientific studies that are now a part of the professional course, say anatomy, physiology, chemistry, biology, are not as valuable towards acquiring the A.B. degree as studies that are purely literary in their character?

Third. Whether the objection to the year spent in a professional school, if spent in any other school, would not be answered by the fact that a man who wants a Harvard degree is expected to pass Harvard examinations? To suppose an extreme case, if a man had spent his three years in Harvard College, and desired to graduate in medicine from Columbia University, he goes to Columbia and spends his fourth year, passes his examination, and graduates. Now, if he wants the Harvard degree, it belongs to him to come back and pass the Harvard examinations; and I ask whether it would be any lack of courtesy towards these institutions, or education at large, to require that? We cannot conceive of Harvard giving degrees on any other institution's examinations, particularly as she will not even admit them to preliminary grades in the college on a certificate of any other examination than her own.

*Professor Macvane.*—As for the first question, gentlemen, I think nobody could answer it; I am sure I cannot, because you would have to get, first of all, a set of men who were examining candidates for admission twenty-five years ago, and are still at the business. This is a matter that cannot be accurately measured by any person, but there are some materials for a judgment about it. We have added some subjects to the requirements: for instance, twenty-five years ago there was no requirement in modern languages. A man came into Harvard without either French or German. Now we require both French and German. And a new requirement in physics is added: I do not remember the date, but not very long ago. That has become, I understand, rather a formidable requirement. Probably in all the subjects there has been some increase in the severity of the tests.

I suppose the surest evidence on the point would be found in the average age of the men who pass, and that has risen, certainly. Whereas fifty years ago men were able to get into Harvard at between sixteen and seventeen, they are now between eighteen and nineteen. For a dozen years, ending with 1897, the average went even above nineteen, but it is below now. Whether the rise of age is wholly due to increasing hardness of the examinations and the addition of new subjects, nobody can tell. I think it is partly due to these things, but I have no doubt it is also partly due to the increase of other attractions to our young men in the schools.

Teachers will tell you that it is increasingly difficult to get things done in the foot-ball season and in other seasons. There are other attractions. Social life in America has become more varied and much more attractive. Young people will not spend so long a time, and their parents will not drive them to spend so long a time, over their books as your fathers and grandfathers did. American parents, in my observation of them, do not want their sons and daughters to work very hard: they do not see the need of it. A mother will take her boy off on a trip to the country, or let him see a foot-ball match, or go to some entertainment, nowadays; when, of old, a mother would say, get down to your geometry or your algebra. The spirit of study in the schools is as good as ever, but the difficulty of getting things done is increasing. So I think the rise of age is partly due to that fact; and there may be other things that influence it. The unmistakable thing is that there has been a rise in the age, very decidedly. It is of late tending downward again, we do not know exactly why. Possibly in Harvard it is due to an increased freedom of choice. We do not now drive anybody to take so great a quantity of prescribed subjects, nor do we require both Latin and Greek. If a boy does not like the ancient languages, he can cut one of them out altogether, and substitute other things. For his so-called "advanced subjects," his special and best work, he has a wide field of choice; so he and his parents and his teachers can consult his native bent and ability. It is found that students will do more of things they choose for themselves than of things we impose upon them; so the greater freedom of study is tending, we hope, to shorten the period of preparation for college.

The question as to the comparative value of scientific and literary studies in training the mind, is one as to which the world has had much debate, and probably will have more. The practical solution adopted by Harvard is to demand mainly a literary training as a preparation for entrance to college, and to give students once admitted almost complete freedom in the choice of studies. In college work scientific and literary studies are put on a basis of equality.

As to the other question, of counting for our degrees work done in other places, my friend, Dr. Fillebrown, is mistaken in one point. We do that already. We admit to Harvard College every year scores and scores of men who have not passed our entrance examinations, but have passed and studied in other colleges; we admit them on their record. Many are thus admitted even to the senior class, and get their Bachelor's degree in one year.

As to the administrative side of this question of counting work done in other colleges, there would be great difficulty in testing it by any Harvard examinations. Our ordinary examinations would not fit their courses. We have not courses corresponding exactly to what they offer at other places, and so we should have to make special examinations for each applicant, which would be a great burden. Further, we are not willing to say that college work consists in passing examinations any way. We try to keep examinations as far as possible in the background, and to insist that men are there for work and not to pass examinations. So we prefer to judge candidates by the rating, and the testimony of their previous college teachers.

*Dr. Fillebrown.*—The third question was, how far purely scientific studies that are taken in a Harvard professional course can be counted towards the fourth year. I believe the matter has been discussed whether physiology and chemistry, and those purely scientific studies, may not come in as a choice for the fourth year.

*Professor Macvane.*—Some of them do. We offer some courses ourselves in the scientific departments that appear substantially to duplicate courses in professional schools. This matter is in the hands of our scientific professors. I think the faculty will admit any scientific work that those men say ought to be admitted to count for the college degree. And what they say, as I remember, is that where you are carrying on a strictly professional course, as in most of the schools, the method is usually narrow and technical, arriving at a specific practical result by the shortest road. It is the old-fashioned way of packing a man's mind full of the facts, or of giving him the mechanical skill that may assure him success in the money making business. That is said by persons who are supposed to know about this work. If you can satisfy our scientific men that the work in any such course is done in the scientific spirit, they will, I think, accept it to any extent for college purposes.

*Dr. Fillebrown.*—That will shorten one course or the other?

*Professor Macvane.*—It may. That is the result in the Harvard Scientific School. A graduate who has taken courses in college that count in the scientific school can get the Bachelor of Science degree in two years; and I do not know but that he could get it in one year after graduating from college.

*Dr. Fillebrown.*—I wish, if the time will serve, the subject might be opened up to the remarks and questions, because it is a matter that is interesting to every man. Others may have questions that they would like to ask.

*Dr. Smith.*—The remarks of Professor Macvane have been doubly interesting to me, inasmuch as my work places me in this atmosphere of education,—dental education, in particular,—and I have, therefore, been somewhat in touch with the problems which he has so ably presented.

It is quite true that the requirements of the professional schools, and especially a majority of dental schools, have been, and are, very low. And I could not help thinking, Mr. President, when you were speaking, and comparing the dentist, or rather, the education of the dentist, with the education of the physician or surgeon, that it was a bit unfair to cite, as many do, the requirements for Johns Hopkins or the Harvard Medical School as the type representing the standing of a majority of the medical schools.

Now, this is not true. A majority of the medical schools are to-day admitting students to medical education on very low requirements, and largely for the reason that the professional schools are generally without endowments, while colleges are well endowed and are not wholly dependent upon receipts from students, as are professional schools.

There is another thing that dentists do not consider sufficiently: they are always comparing the attainments of the dental profession, which is a very young profession, with the attainments of the medical profession, which is a very old profession. They should compare the standing of the medical profession at the close of its first forty years with the standing of the dental profession during a like period.

This matter of dental education presents itself in two ways: first, the preliminary requirements; second, the degree which the dentist should receive. On the one hand we have advocates of the degree in arts or letters for the student who enters the dental school; on the other hand, in addition to this, we have the advocates of the medical degree for the man who is to become a dentist. Now, if we adopt both of these requirements, what does it mean? If the college course is to be four years, and the student does not enter college until about eighteen, he is twenty-two years of age before he receives his Bachelor of Arts degree. If he receives a medical education, that means four years more, and brings him to the age of twenty-six. And then, if he is to be any kind of a dentist, he certainly needs, in addition, two years' study in a dental school in subjects applicable to the specialty of dentistry, including much technical training. That makes him twenty-eight years of age before he goes out into the world to practise,—too late in life, I think, for a man to begin his professional work.

For my part, I have been a believer and have used what influence I could to bring about the higher preliminary requirements for students entering the dental profession; believing that the medical degree, as given to-day, is not at all necessary for a dentist. It is quite true that there are a great many medical subjects which he needs, and which he gets in the better dental schools. It is the purpose (if I may be allowed to speak for the Harvard school, in which I am interested) to bring its preliminary requirements as fast as possible up to the A.B. degree, making it a graduate department of the University. And, looking to this end, we announce in our present catalogue that in June, 1904, and thereafter, examinations in English, physics, either Latin or French, and chemistry will be obligatory, and also an elective of either German, botany, geometry, or shop work, such as is given in the Lawrence Scientific School (believing that the dentist should have these technical requirements). These examinations are to be of the same grade as like examinations to Harvard College, and to be taken at the same time and places. This, I think I may say without any boasting, is a step in advance of other dental schools.

*Dr. Potter.*—I want to express my appreciation of the way in which the subject has been presented to us this evening by Professor Macvane. It seems to me the most important subject that we can consider at the present time.

I have long held the opinion which Dr. Smith has expressed, that what dental students need most of all is adequate preliminary education. Recently I read in a German dental magazine a long report of a committee appointed to investigate and report upon the American dental schools. This committee had come to this country and had looked into all the dental schools, and they made an extensive report as to their condition. The main criticism made by the committee was that the preliminary qualifications for entrance to the dental schools were entirely inadequate. And it made this further remark: "Of course the Americans are very skilful in practical matters; they are not sufficiently educated to busy themselves about purely scientific matters." And that is partly true.

Dr. Fillebrown asked a question which perhaps I can answer. He asked how the entrance examinations at Harvard College differ at the present time from those held twenty-five years ago. I took my entrance examination to Harvard College more than twenty years ago, and I know something of their difficulty at that time. During the last year I have had occasion to examine the most recent entrance examinations, and I am sure that they are more difficult than those of twenty years ago. There are additional subjects, as Professor Macvane has said; but, more than that, the individual examinations are more difficult, and it requires a better trained man to pass the examinations now than it did in my time, although we thought at that time the examinations were quite severe.

I am very glad this subject has been brought up. It seems to me the most profitable and instructive subject we have had at an annual meeting for a long time. I believe when we discuss matters of liberal education we get at the root of progress in dental education.

*Dr. Fillebrown.*—Just one more question. How much time does this advance represent? How much longer does it take a man to fit for Harvard College to-day than it did twenty-three years ago?

*Dr. Potter.*—As I remember, when I entered college the average age was eighteen. Professor Macvane says it is now a trifle over eighteen.

*Dr. Fillebrown.*—I do not think that would answer my question. My question is, no matter whether a man is forty years of age or fifteen; if he could pass the examinations that you passed twenty-three or twenty-four years ago, how much longer would that same man have to study to prepare himself for the examinations to-day? That is what I mean, without reference to his age.

*Dr. Potter.*—That is rather difficult. I suppose methods for fitting men have improved. Very likely they can fit men just as quickly now for the harder examinations as before for those not so severe.

*Dr. Fillebrown.*—What I was after is the time element, as there is so much said at the present time about its taking a man so long and his getting so old before he enters his profession, commences his life work. I wanted to get some idea of how much more of a man's life it is taking to prepare for college now than in former years, or if it can be done in the same time.

While I am on my feet, I want to refer to the statement that was made in regard to the medical degree for dentists. The position of those who advocate that degree is not generally accurately stated, and it was not quite acurately stated to-night. I do not mean not fairly stated, but not accurately stated. We all know that President Eliot a few years ago at the alumni meeting of the Medical School advanced the position that the condition of medicine to-day is similar to the condition of literary studies at Cambridge. There they have a series of courses that, if one man took all of them, it would take him about twenty years to complete them. Yet from all that mass of courses a man may select a course that will lead him to his Bachelor of Arts degree in four years; or, in an exceptional case, in three years. President Eliot went on and stated that the science of medicine at the present time covered so wide a field that if a man should complete his studies in all the branches, it would take him at least twelve years to do it. He said Harvard University to-day ought to offer courses in medicine that it would take a man a good twelve years to complete and become fitted in them. Few men can afford to spend that time. There ought to be a series of courses presented that lead to the M.D. degree, that a man could complete in four years and know something nearly complete about some branch of medicine that he wants to practise. It is no longer possible for one man to do everything, all the way from dentistry to obstetrics.

I am one of those that for twenty-five years or more have persistently, in season and out of season, advocated the idea of a medical degree for dentists, that dentistry is medicine as much as removing the fæces from an impacted anus, or a piece of wax from an ear. Now, I say that dentistry is essentially a medical occupation, and the dentist is entitled to have the same standing as the surgeon, the oculist, the aurist, or any other of the medical specialists. I say that the practical part of medicine is as much a part of the medical course as is the theoretical. It is as essential to know how to apply medicine as to extemporize a splint for a broken limb in the wilderness. That is a part of the requirements for his degree. So it should be a part of the requirements of the medical degree that a man should know something about dentistry. He must know whether he is treating a simple abscess of the jaw, or a necrosed bone or a cancer. I have had case after case where patients had been operated upon for necrosis of the jaw, when all the trouble with them was a piece of tooth root left in the jaw, which was keeping up a continual discharge from an abscess.

The thing is to make the dental course one of the medical courses. That is, the practical part of the dental art shall come in as a substitute for some other course, and so educate the man and make him a practical dental specialist. Medical students ought to be made to know something about the treatment of diseases of the jaw dependent upon the teeth. So, when the medical degree for dentists is being represented, I hope it will always be borne in mind that that is what it means. It does not mean that practical dentistry shall be sacrificed, but that it is to be substituted; that is all.

*Dr. Smith.*—I was talking with my neighbor, Dr. Banfield, when Professor Fillebrown opened his remarks, but I think he said something about my not stating something fairly. It seems to me that the logic of Professor Fillebrown's argument is that the medical man needs a dental education, rather than the dental man a medical education. I have always had the highest regard for the men in the past who took the dental degree, when it meant but very little, for eking out what they did not get in dental education in getting the medical degree.

Now, for the benefit of some of you who cannot know what a medical degree meant forty years ago in the Harvard Medical School, let me relate it. Not more than thirty years ago a student entered the Harvard Medical School without any preliminary examination whatever. Many of the students could scarcely write a legible hand. The course was two terms of six months each, and the second term was a repetition of the first term. Bacteriology, histology, pathology, physiological chemistry, and comparative medicine were comparatively unknown. At the end of the second term the applicants for the degree went before their professors for examination,—six professors, I think; and, after an oral examination, a majority vote decided the fate of the applicant. Dentistry, even at its worst in educational matters, was never quite so bad as that.

Unless one is in touch with the educational methods of to-day, they have not the remotest idea what a medical education means. This elective scheme in medical education, which Professor Fillebrown emphasizes to-night, is, it seems to me, very much limited in its application, and I find that the medical educators find it so themselves. In college a student must cover eighteen courses for the Bachelor of Arts degree. A and B may start side by side for that degree, and take almost entirely different routes, and both receive the Bachelor of Arts degree. That degree stands for a certain amount of general mental training, while with professional education there must be exact training for a specified practical purpose.

I admit that dentistry is a specialty of medicine, and we need much of medical training, but not all, and the question is where to cut down the courses in our present high standard medical schools and adapt them to our needs as dentists. I know from observation that a man wishing to become a dentist needs, after receiving his medical degree, two years of training in subjects that directly deal with dentistry. I have observed that many men who hold the degree in arts and the degree in medicine, are yet unable to pass the examinations and the requirements of the dental school in less than two years. I believe that with the immense technical requirements upon a dentist of to-day, he has got to curtail in his education somewhere; and I am not one who advocates the curtailing in the preliminary requirements.

*Dr. Fillebrown.*—We are soon, no doubt, to have a four years' course in our own school, an equivalent in time with the medical school. Now, when the time comes that medicine is taught as I believe it should be, and men are graduated competent to practise the various specialties which they want to pursue, the first two years are going to be devoted to subjects which we all admit are good for use in any practice whatever. These two years will cover more medicine than was covered in the medical schools only a few years ago, a great deal more than is given in any dental school to-day. Then the other two years will be left for the acquisition of the practical part of dentistry. They will also be given to the acquisition of the practical part of each other specialty, whichever a man chooses. The general practitioner will then become a specialist. The man who wants to go into the country and practise becomes a specialist, because no man can fit himself as a general practitioner and still be up in all the various specialties and do the finer work in surgery and the finer work in the eye and ear or in any one of the specialties that are practised to-day. It is now the practice of most all the general practitioners to send patients to the medical centres for special operations.

Now then, you say that if this system is to be adopted, a man will wander around from one specialty to another after he gets his degree and finally settle on something that he will like. To-day if a man wants to graduate in medicine he has to spend two or three years or more on his specialty after graduation, perhaps as much time as he spent in his special course. I say, when a man graduates from the four years' course, he ought to be fairly expert in one department. A man cannot practice and cannot fit himself in a lifetime in all the specialties that are offered to-day. To-day the medical degree represents every specialty that is now practised, except the one of dentistry, and why should that be left outside?

*Dr. Williams.*—Perhaps a little history in this matter would be appropriate. After a long illness, which interrupted my college life, I was advised to study medicine, but was told that I would not be strong enough to practise medicine, with its exposures and hardships, and was again advised that I had better take some specialty,—get a chance to study dentistry, perhaps.

In Boston, Dr. Harwood, Dr. Flagg, Dr. Keep, and Dr. Tucker were the principal dentists then, and none of them would take a student unless he would agree to go through a medical course, and understand the general principles in order to know how to apply them to his specialty. I went into the Harvard School (Tremont School they then called it), in which the professors taught classes: Dr. Holmes was one, Dr. Bigelow, Dr. Ware, and several others. I also attended the winter courses. Then only three years were required. I took four years for the whole before I graduated and took my medical degree.

Those medical courses were very simple, but they did not leave the general principles of medicine for the minutiæ of specialties, and time was taken for investigation. They were taught by practitioners of medicine, generally of experience, enough of the general principles of medicine to know what effect carious ailments would have on the welfare of the general system. That school graduated some noble men, like Henry J. Bigelow, our present Dr. Cheever, Hodges, Charles Homans, and several other very noted men who were grounded in the general principles of medicine and surgery. They knew how to apply what knowledge of medical principles they had learned to their specialty. The oculists also got a knowledge of general principles. Like an agriculturist, for instance; one may want to cultivate flowers and fruits, but he learns the general principles of agriculture, of climate, soil, etc., and he knows how to apply that knowledge to his particular line. So it has been applied in this way. And, in fact, that is the way nature constructed the science of medicine; all the parts of the system to harmonize with the other parts in health, and to sympathize with the other parts in disease. So that, for the greatest success, one must have this knowledge, whatever the degree may be. The title sometimes amounts to nothing in practical use; for I have known men without the medical degree who have studied up, and who catch principles by hints rather than by hard studying, who were as well versed in knowledge of principles as some who have been through the course and got their degree.

For the lack of that knowledge I have seen a great many failures with some most skilful operations. I have seen several cases of fatal results from that lack, and I can tell you one. A noted preacher, not far from here, a few years ago had trouble with four of his upper incisors wearing down so that he thought his articulation was affected. And a man in the neighborhood, practising in cohesive gold architecture and contour work, boasted that he could build gold to any extent. They said he was a great "blower," and he blew so hard as to reach the ears of this clergyman, who went to him, and he hammered on that patient's teeth four or five hours a day for five consecutive days. I remember in my medical course the elder Aggasiz used to tell us in his classes how a continued comparatively slight irritation of the brain could produce a shock of paralysis on the brain equal to the blow of a policeman's club. This hammering on this patient's teeth produced the same effect. This man had jumped from some mechanical work to dentistry, and as a blacksmith hammers on his work, so he hammered on the patient's teeth, who thought he must bear it, that a man must be superior to physical pain; and at the end of that period he was taken with a paralytic shock, from which he partially recovered and lingered for two or three years, and died. There was a lack of medical knowledge in the operator.

I could tell you of two or three other cases of too large a number of teeth being taken out at one time, producing such a shock that a person sank gradually, faded away, and died in two or three weeks. I remember my old preceptor never used to take out more than two teeth, on account of the great strain on the system required to heal a certain surface of the gum. A much greater vital force is required to heal a surface of that sort than a larger surface on the skin. That shock and strain on the system caused death in two instances from a lack of medical knowledge in the dentist giving the patient more to bear than the constitution would stand; if he had taken a general view of the patient's system and constitution, knowing the possibilities and the sympathies, he would have refrained. I could enumerate several cases of the sort.

The point is, in this matter of naming degrees, etc., they simply represent a supposed knowledge; but there are people who have that knowledge without the degree. But of course, if you want to get a degree, the only way is to go where you will get the best,—*i.e.*, to a college of proper standing. That is one point, I think, that has not been sufficiently considered in all this talk about the education in specialties, that they must not be followed out, any of them, in its line without keeping in mind the relation to the general health and the general system, and the general effect of these special lines on the main system. That is what I want to impress. The idea is just here: Dr. Fillebrown's question has often occurred to me, whether in the medical course on the elective plan some of the time might not be devoted to some specialty by the pupil, and so properly count on his medical education. It seems to me quite proper, especially if the first part of his medical course has been so thorough in general principles that he is enabled to apply those principles to a specialty.

There is another thing: in our dental schools formerly (it is much better now) students were educated by rote. The teacher came before the class with the idea that if certain things were so and so, certain things were done so and so. The point is that the great excellence in any specialty is that in technical education, which is important in any specialty, it should be accompanied and followed under knowledge of the general principles applying to that profession.

*Dr. Meriam.*—I do not like the idea of the college losing its position and becoming merely a fitting for special schools. It should be complete in itself. Parents have a right to draw back if the question arises, if, in sending a boy to college, they are to send him to one that is planned to be secondary to such schools. If from this the young men of to-day must be carried by the fathers until they are nearly thirty, it is more of a burden than should be placed on American life.

*President Pond.*—Gentlemen, I think Professor Macvane can feel that he made a very happy selection of his subject; certainly it has proved a very interesting one, and it has proved a subject that has brought out our thoughts. Is there anything more to be said on the subject?

*Professor Macvane.*—If you will excuse me, just a word. I was not sure, any more than the last speaker was himself, just where he was leading us. As to that one point, however, that the college tends to become a servant of the professional schools, there is something in that, undoubtedly, but perhaps not so very much, for whatever is counted now in Harvard College, so far as I know, towards the A.B. degree is certified by the experts in that field as a liberal study. It may or may not turn out to be valuable in a professional way later. It is not primarily conducted for professional ends. For instance, when you take a course in biology, it is certified to the faculty as a research into pure science. It is training in the knowledge of nature. It is true that that training may turn out later to be immensely valuable in the study of medicine, but in our use of it it is good for everybody; it is liberal education. The engineering department has grown wonderfully. You will find, in the vast array of engineering courses, only a part counts towards the A.B. degree. Those we count are courses of broad culture, rather than strictly professional courses. Where they tell us this is a strictly professional course for the engineer, we say that it cannot be counted for the A.B. degree. I do not know that we are right in all cases, but we have at least a clear principle.

*Dr. Bracket.*—I have been especially gratified in being present to-night, and feel that there has been said by many speakers a great deal that has been interesting, profitable, and suggestive for us to hear. Out of this annual meeting there should come much good. The ideas of all of us will be broader, more comprehensive, and more practical than they have been before.

We are under especial obligation to the gentleman who has introduced the discussion this evening, and has spoken from a broad and liberal stand-point. I move that the thanks of the Academy be presented to Professor Macvane for his effort before us.

Voted.

*President Pond.*—I want to thank the Academy once more for the great honor they have given me. I am sure I appreciate it very much. I want to thank you also for the very great kindness shown and the easy manner in which you have made it possible for me to perform my duties.

I believe it is one of my pleasant duties to present our new President, and I would like to ask Dr. Cooke and Dr. Boardman to escort him to this end of the room.

It gives me great pleasure to present Dr. Bradley.

*Dr. Bradley.*—Fellows of the American Academy of Dental Science, at this hour of the evening extended remarks from me would be superfluous. Nevertheless, a suitable recognition of the honor you have conferred on me may be in order.

I have always appreciated fellowship in the Academy very greatly, and have felt that with the privileges of fellowship come also the duty to serve and minister to the success of the organization in every way that I could. In the position to which you have elected me, it will still be my desire to serve you to the best of my ability.

Fellows of the Academy, I thank you for the honor conferred, and await your pleasure.

*Dr. Smith.*—With the president whose term of office expires, three men retire from office this year,—Drs. Pond, Perrin, and Payne. The committee tried in every way to have Drs. Perrin and Payne continue in service. They have both served the Academy faithfully and well for a number of years, and evinced a strong desire to be relieved of the cares and duties attending their respective positions. So I move you, Mr. President, that a vote of thanks be extended to the president and the other retiring officers, for their efficient and able service.

Voted.

On motion, adjourned.

CHARLES H. TAFT,
*Editor American Academy of Dental Science.*

